# Supplementary material for: Comparative genomics provides new insights into the diversity, physiology, and sexuality of the only industrially exploited tremellomycete: Phaffia rhodozyma
Source: BMC Genomics. 2016 Nov 9;17:901. doi: 10.1186/s12864-016-3244-7 (PMC5103461; doi:10.1186/s12864-016-3244-7)
Supplement: Additional file 6: — List of orphan genes with links to PFAM (related to Additional file 1: Table S1). (ZIP 1428 kb) [file 12864_2016_3244_MOESM6_ESM.zip › PFAM_UNIQUE/G00577_P.html]

#                                                                            --- full sequence --- -------------- this domain -------------   hmm coord   ali coord   env coord
  
# target name        accession   tlen query name           accession   qlen   E-value  score  bias   #  of  c-Evalue  i-Evalue  score  bias  from    to  from    to  from    to  acc description of target
  
#------------------- ---------- ----- -------------------- ---------- ----- --------- ------ ----- --- --- --------- --------- ------ ----- ----- ----- ----- ----- ----- ----- ---- ---------------------
  
G00577\_P             -            861 AIB                  PF15334.1    324       7.7    4.1   7.6   1   2   0.00049      0.98    7.1   1.5    14   100   107   202   103   224 0.74 - Aurora kinase A and ninein interacting protein  
G00577\_P             -            861 AIB                  PF15334.1    324       7.7    4.1   7.6   2   2      0.17   3.4e+02   -1.3   0.5   259   289   554   583   477   608 0.59 - Aurora kinase A and ninein interacting protein
